# Real-World Data of Comprehensive Genomic Profiles and Clinicopathological Characteristics of Duodenal Epithelial Neoplasms

**DOI:** 10.3390/cancers18132097

**Published:** 2026-06-28

**Authors:** Marin Ishikawa, Hideyuki Hayashi, Kohei Nakamura, Ryutaro Kawano, Eriko Aimono, Hiroshi Nishihara

**Affiliations:** 1 Center for Preventive Medicine, Keio University School of Medicine, Azabudai Hills Mori JP Tower 7F, 1-3-1, Azabudai, Minato-ku, Tokyo 106-0041, Japan; 2 Cancer Center, Keio University School of Medicine, 35, Shinanomachi, Shinjuku-ku, Tokyo 160-8582, Japan; rock-hayashi-pop@keio.jp; 3 Center for Cancer Genomics, Keio University School of Medicine, 35, Shinanomachi, Shinjuku-ku, Tokyo 160-8582, Japan; knakamura320@keio.jp (K.N.); ryu.kawano@keio.jp (R.K.); hnishihara1971@keio.jp (H.N.); 4 LUMIPATH Clinic, 2-2-501, Kita 5-jo Nishi 12-Chome, Chuo-ku, Sapporo 060-0005, Hokkaido, Japan; aimono@lumipath-clinic.com

**Keywords:** duodenal epithelial neoplasms, genomic profile, mucin phenotype, tumor mutational burden

## Abstract

**Background/Objectives**: Duodenal epithelial neoplasms are rare; however, the widespread use of surveillance endoscopy and advances in endoscopic imaging technology have increased their incidental detection. Owing to their rarity, the clinicopathological characteristics and natural course of duodenal epithelial neoplasms have not been thoroughly investigated. In this study, we aimed to clarify the genomic profile and clinicopathological characteristics of duodenal epithelial neoplasms. **Methods**: A total of 158 patients with duodenal epithelial neoplasms were enrolled. Comprehensive genomic profiling and immunohistochemical staining were performed. Immunophenotypes were categorized as gastric type (G-type), gastrointestinal type (GI-type), or intestinal type (I-type). The detection rate of potentially actionable genomic alterations and a high tumor mutational burden (TMB-H ≥ 10 Muts/Mb) were evaluated and compared across tumor types. **Results**: The median size of adenocarcinomas was larger than that of adenomas (*p* = 0.002). The age at diagnosis of G-type tumors was higher than that for the other two tumor types (*p* < 0.001). The median size of I-type tumors was smaller than that of the other two tumor types (*p* = 0.019). Compared with the other two types, G-type tumors were predominantly located in the superior region (*p* < 0.001), were macroscopic Type I (*p* = 0.002), and had significantly higher genomic alteration rates for *KRAS* (*p* < 0.001), *GNAS* (*p* < 0.001), *CDKN2A* (*p* = 0.004), and *MDM2* (*p* < 0.001). Eighteen patients showed TMB-H. **Conclusions**: TMB-H was observed in >10% duodenal tumors. Additionally, the pathogenesis of G-type duodenal tumors differs from that of other immunophenotypic tumors. These findings could help in understanding the genomic profiles of duodenal tumors and in selecting treatment options.

## Simple Summary

Duodenal epithelial neoplasms are rare; therefore, their clinicopathological characteristics and natural course have not been thoroughly investigated. Here, we performed immunohistochemical staining and genomic analysis of 158 cases of duodenal epithelial neoplasms and analyzed their clinicopathological characteristics and genomic profiles. This approach revealed that the immunophenotype, particularly in gastric-type tumors, is significantly associated with mutations in *KRAS*, *GNAS*, *CDKN2A*, and *MDM2*. On the basis of the genomic profiles of each tumor type identified in this study, as well as previously reported findings, we have proposed a mechanism for duodenal epithelial neoplasm carcinogenesis. We believe that our findings contribute to the elucidation of various mechanisms underlying the development of duodenal tumors.

## 1. Introduction

In 2022, the incidence of small intestinal cancer, classified as a rare cancer, was reported to be 2.6 per 100,000 people [1]. However, the overall incidence of small intestinal cancer has been slowly increasing from the early 1990s [2,3]. The most common histological type of small intestinal cancer is adenocarcinoma, accounting for 31–40% of all small intestinal cancers [3], followed by neuroendocrine tumors, lymphomas, and sarcomas (mostly commonly gastrointestinal stromal tumors and leiomyosarcomas). Most cases of adenocarcinoma occur in the duodenum (60%), with 30% occurring in the jejunum, and 10% in the ileum [4,5,6]. The median age at diagnosis is approximately 60 years, with a higher incidence among men than among women [3,5,6,7].

In addition, duodenal adenomas are rare lesions, with a prevalence of <0.1–0.3% among patients undergoing upper gastrointestinal endoscopy [8,9]. However, with the widespread use of surveillance endoscopy and advances in endoscopic imaging technology, the incidental detection of these lesions has increased [10]. The adenoma–carcinoma sequence [11,12,13], one of the mechanisms underlying colorectal cancer (CRC) development, is considered a possible pathogenic mechanism for duodenal adenocarcinoma. However, owing to their rarity, the clinicopathological characteristics and natural course of these lesions have not been thoroughly investigated.

Genomic analyses of small intestinal cancers have been reported, and their molecular characteristics have been described. In 2021, a study analyzed 24 cases of small intestinal adenocarcinoma using the FOUNDATION Cdx technology and classified them into three subtypes [14]. Additionally, small intestinal adenocarcinoma has been reported to harbor a high frequency of mutations in *TP53*, *KRAS*, *APC*, and *SMAD4* [15,16,17,18,19,20,21,22,23,24,25] and to share some similarities with CRC. However, analyses comparing gastric cancers, small intestinal cancers and CRCs have also reported that small intestinal cancers exhibit characteristics distinct from those of gastric cancers and CRCs [19]. The small intestine is approximately 6–7 m in length; consequently, small intestinal cancer has been reported to exhibit molecular differences depending on the site of origin. Duodenal cancer has a higher frequency of *ERBB2* alterations and a lower frequency of *CDKN2A* alterations than does jejunal/ileal cancer [19]. By contrast, jejunal/ileal cancer has a higher frequency of tumor mutational burden-high (TMB-H), microsatellite instability-high (MSI-H), and *APC* and *POLE* mutations [25].

Several studies have investigated the molecular and clinicopathological characteristics of duodenal tumors. Miyamoto et al. [26] performed genomic profiling of intestinal/mixed-type superficial non-papillary duodenal epithelial tumors, elucidating their genetic characteristics. This study specifically analyzed major genomic alterations, such as *APC*. Fukusada et al. [27] examined the relationship between genomic alterations and clinicopathological features in non-papillary duodenal epithelial tumors. This study indicated that specific genomic alterations correlate with tumor malignancy and morphological characteristics. Kinugasa et al. [28] investigated the impact of *KRAS* mutations in patients with sporadic non-papillary duodenal epithelial tumors, discussing their effects on tumor progression and clinical behavior. Hida et al. [29] conducted immunohistochemical and genomic analyses of gastric type (G-type) duodenal tumors and proposed a practical classification system. A key feature of this study was elucidating the characteristics of G-type phenotype tumors. Kim et al. [30] performed clinicopathological analyses and survival analyses of duodenal adenocarcinoma, reporting on patient prognosis and associated factors. Yoshida et al. [31] examined the clinicopathological features and phenotypic classification of non-papillary duodenal tumors, elucidating the characteristics and clinical differences among phenotypes. These previous studies have yielded important insights into the genomic background, clinicopathological features, carcinogenic pathways, and differences between subtypes of duodenal epithelial tumors. However, unresolved issues remain, including the detailed mechanisms of carcinogenesis across subtypes, specific impact of molecular abnormalities on clinical course, and establishment of optimal treatment strategies. The clinical importance of subtype-specific genomic alterations and the establishment of prognosis prediction and personalized treatment strategies based on them remain ongoing challenges.

In this study, we aimed to clarify the genomic profiles and clinicopathological characteristics of duodenal tumors. The results of this study are expected to contribute to elucidating the mechanism underlying duodenal tumor development.

## 2. Materials and Methods

### 2.1. Patients

This study included patients who underwent duodenal tumor resection at Keio University Hospital between December 2018 and February 2024. Inclusion criteria were histopathological diagnosis of duodenal epithelial tumor, completion of comprehensive genomic profiling testing at the hospital in clinical practice, and age ≥ 18 years at the time of testing. Exclusion criteria were duodenal papillary tumors and patient refusal to participate in the study. An opt-out document was posted on the hospital website, explicitly informing eligible patients of their right to refuse participation. No refusals were recorded, resulting in 158 patients being included in the study. Clinical information (tumor location, macroscopic findings, histopathological type, resection method, etc.) was retrospectively extracted from medical records. This study was approved by the Keio University School of Medicine Ethics Committee (approval number: 2024-1064). It was conducted in accordance with the ethical standards set forth in the 1964 Declaration of Helsinki and the Ethical Guidelines for Medical and Health Research Involving Human Subjects.

### 2.2. Histological Analysis

The histological findings of all specimens were reviewed by two independent pathologists who were blinded to clinical and molecular information. The immunophenotype was evaluated based on results of MUC5AC, MUC6, MUC2, and CD10 immunostaining.

### 2.3. Immunohistochemical (IHC) Staining

IHC was performed to determine the immunophenotype. Consecutive 4 µm thick sections were deparaffinized and dehydrated through a graded series of xylene and ethanol solutions, respectively. Each section was subjected to EnVisionTM flex target retrieval solution (high pH; DAKO, Carpinteria, CA, USA) for 20 min at 95 °C for activation, and an automated stainer (Nichirei Biosciences, Tokyo, Japan) was used according to the vendor’s protocol. IHC was performed using primary rabbit antibodies against MUC6 (ab223846; 1:1500 dilution; Abcam, Cambridge, UK) and primary mouse antibodies against MUC5AC (NCL-MUC-5AC; 1:100 dilution; Leica Biosystems, Newcastle upon Tyne, UK), CD10 (NCL-L-CD10-270; 1:100 dilution; Leica Biosystems), and MUC2 (#88686; 1:500 dilution; Cell Signaling Technology, Danvers, MA, USA), followed by a secondary antibody (Histofine Simple Stain MAX-PO (MULTI); Nichirei Biosciences). All immunostained specimens were assessed by an independent observer blinded to clinical information.

The staining intensity was classified as negative (0), weak (1+), moderate (2+), or strong (3+). When >10% tumor cells were stained with ≥2+ intensity, the tissue was considered positive [27]. The immunophenotype was classified into three types: gastric type (G-type) (MUC5AC- and/or MUC6-positive but neither MUC2- nor CD10-positive), gastrointestinal type (GI-type) (MUC5AC- and/or MUC6-positive and MUC2- and/or CD10-positive), or intestinal type (I-type) (MUC2- and/or CD10-positive but not MUC5AC- nor MUC6-positive). Figure S1 presents representative examples of each immunophenotype of duodenal epithelial neoplasms. Samples that were negative for these markers were not classified.

### 2.4. DNA Isolation

DNA was isolated from formalin-fixed, paraffin-embedded (FFPE) sections using the Maxwell RCS DNA FFPE kit AS1450 (Promega, Madison, WI, USA) from 2018 to 2022 and the Maxwell RSC FFPE Plus DNA kit AS1720 (Promega) from 2023 to 2024. The Qubit 158dsDNA BR Assay Kit and Qubit 4 (Invitrogen, Waltham, MA, USA) were used to quantify the concentration of the purified DNA. A genomic DNA screen tape, genomic DNA Reagents, and a TapeStation 4200 (Agilent Technologies, Santa Clara, CA, USA) were used to quantify the DNA integrity number (DIN).

### 2.5. Sequencing Analysis

From 2018 to 2022, 108 samples were sequenced in the PleSSison-Rapid test using GeneRead DNAseq Targeted Panel V2 Human Comprehensive (Qiagen, Hilden, Germany), and from 2023 to 2024, 50 samples were sequenced in the Rapid-Neo test using the SureSelect PrePool Custom Tier2 (0.5–2.9 Mb), 96 hyb (Agilent Technologies). Targeted amplicon sequencing of 160 cancer-related genes was performed using the GeneRead DNAseq Targeted Panel V2 Human Comprehensive (see Table S1: List of 160 cancer-related genes in the PleSSison-Rapid test). By contrast, the SureSelect PrePool Custom Tier2 was a customized panel encompassing all exons of 145 cancer-related genes and 11 fusion targets (see Table S2: List of 145 cancer-related genes and 11 fusions in the Rapid-Neo test). The templates were sequenced using the NextSeq 550 (Illumina, San Diego, CA, USA) and the NextSeq 500/550 Mid Output kit (300 cycles) (Illumina) following polymerase chain reaction (PCR). We analyzed nearly all exonic regions of the genes common to both panels, and our statistical analysis focused on those genes.

### 2.6. Identification of Genomic Alterations

The presence of genomic alterations, including base substitutions, indels, fusions/rearrangements, and copy number alterations (CNAs), was evaluated. Although corrected to some extent by tumor content, a variant allele frequency (VAF) of 4% was established as the cut-off value. Each genomic alteration was evaluated using the original scoring system (Figure S2: Scoring system of genomic alteration) that we previously reported [32]. The score was based on the following factors: the population of carcinoma clones, function of gene alterations (using reference databases, such as COSMIC, ClinVar, OncoKB, CIViC, and JAX CKB), and the effect of CNAs.

Potentially actionable genomic alterations were defined as those with a biological significance score of ≥2 and no variants of unknown significance (VUS). TMB-H was defined as the presence of ≥10 Muts/Mb. The detection rates of potentially actionable genomic alterations and TMB-H were evaluated for each type of duodenal tumor.

### 2.7. Statistical Analysis

All categorical variables are expressed as number (percentage), while continuous parameters are expressed as mean [range]. The mean values between two or more independent groups were compared using the Mann–Whitney U or Kruskal–Wallis test. Categorical data between two or more groups were compared using the Fisher exact test. All statistical analyses were performed using SPSS Statistics (version 25; International Business Machines Co., Armonk, NY, USA), and *p*-values < 0.05 were considered statistically significant.

## 3. Results

### 3.1. Clinicopathological and Genomic Characteristics of 158 Duodenal Epithelial Neoplasms

There were 104 male (66%) and 54 female (34%) patients, and the mean age at diagnosis was 67.0 years (95% confidence interval [CI], 65.2 to 68.7). The mean tumor size was 29.8 mm (95% CI, 26.3–33.2), and the resection method was primarily endoscopic (97%). The primary tumor sites were as follows: superior (*n* = 41, 26%), descending (*n* = 77, 49%), and horizontal (*n* = 40, 25%). Among macroscopic classifications, Type IIa (*n* = 111, 70%) was the most common, followed by Type I (*n* = 32, 20%), Type IIc (*n* = 8, 5%), Type IIa+IIc (*n* = 4, 2%, Type I + IIb; *n* = 1, 1%), Type 2 (*n* = 1, 1%), and Type 3 (*n* = 1, 1%). Of these, 127 (80%) were adenocarcinomas, and 31 (20%) were adenomas. The immunophenotypes were G-type (*n* = 23, 15%), GI-type (*n* = 83, 52%), and I-type (*n* = 52, 33%). Figure S3 presents the genomic alteration plots of the 158 duodenal epithelial neoplasms. Eighteen cases (11%) showed TMB-H. The genes with alterations were as follows: *APC* mutation (*n* = 73, 46%), *KRAS* mutation (*n* = 50, 32%), *GNAS* mutation (*n* = 17, 11%), *ERBB2* mutation (*n* = 9, 6%), *CDKN2A* loss (*n* = 8, 5%), *ARID1A* mutation (*n* = 7, 4%), *MDM2* amplification (*n* = 7, 4%), and *BRAF* mutation (*n* = 5, 3%).

### 3.2. Clinicopathological and Genomic Characteristics by Histopathological Type

The clinicopathological and genomic characteristics according to histopathological type are shown in Table 1, and genomic alteration plots by histopathological type are presented in Figure S4. No differences in sex or age were observed between the adenocarcinoma and adenoma groups. The mean size of the adenocarcinomas was 31.8 mm (95% CI, 27.9–35.6), which was significantly larger than that of the adenomas (21.7 mm, 95% CI, 13.9–29.6; *p* = 0.002). No differences in the resection method, location, macroscopic classification, or immunophenotype were observed between the adenocarcinoma and adenoma groups. Furthermore, no significant differences in genomic alterations were observed.

The clinicopathological and genomic characteristics of the 127 adenocarcinomas are presented in Table S3. Most adenocarcinomas were early-stage cancers confined to the mucosal layer (94%); only two cases were advanced cancers. Most adenocarcinomas were negative for lymphatic (97%) or venous (98%) invasion.

### 3.3. Clinicopathological and Genomic Characteristics by Immunophenotype

**Figure 1:**
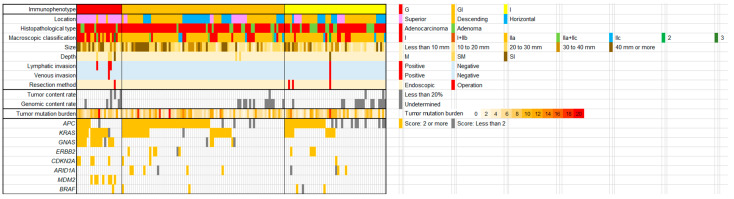
Genomic alteration plots obtained by immunophenotyping. The immunophenotype, location, histopathological type, macroscopic classification, size, depth, lymphatic invasion, venous invasion, resection method, tumor content rate, genomic content rate, and tumor mutational burden are listed in the upper row. The gene names are listed on the left. G, gastric type; GI, gastrointestinal type; I, intestinal type; M, mucosal layer; SM, submucosal layer; SI, serosal invasion.

The clinicopathological and genomic characteristics according to the immunophenotype are shown in Table 2 and Figure 1. No differences in sex were observed among the three types; however, the mean age at diagnosis of G-type was 75.3 years (95% CI, 71.1–79.3), which was higher than that of the other two types (*p* < 0.001). The mean size of I-type tumors was 22.9 mm (95% CI, 17.1–28.7), which was smaller than that of the other two types (*p* = 0.019). No differences were observed between the resection methods. G-type tumors were predominantly located in the superior region (*p* < 0.001), and macroscopic G-type tumors were predominantly type I (*p* = 0.002). No differences in histopathological type were observed. Furthermore, no differences in TMB-H were observed among the three types; however, G-type tumors showed significantly higher alteration rates for *KRAS* (*p* < 0.001), *GNAS* (*p* < 0.001), *CDKN2A* (*p* = 0.004), and *MDM2* (*p* < 0.001) than did the other two types.

## 4. Discussion

This study elucidated the clinicopathological characteristics of G-type tumors. Compared with GI-type and I-type tumors, G-type tumors exhibited distinct genomic alteration profiles. The genomic pathological features of early-stage duodenal tumors were clarified. Furthermore, it was revealed that >10% duodenal tumors exhibit TMB-H.

In the present study, G-type tumors developed proximal region from the papilla of Vater, particularly in the superior region. Similar findings have been reported previously. Watari et al. reported that G-type non-ampullary duodenal adenocarcinomas are frequently observed in the first portion of the duodenum [33]. Hijikata et al. reported that all G-type adenomas are located in the first portion of the duodenum [34].

In the duodenum, the development of G-type tumors requires genomic alterations that transform the intestinal epithelium. Compared with other types of tumors, G-type tumors frequently exhibit *GNAS* alterations and have distinct genomic alterations. Previous reports have documented *GNAS* alterations in gastric metaplasia and G-type tumors [27,35,36]. In this study, *GNAS* alterations were frequently observed in G-type tumors. As no significant difference was found in the incidence of *GNAS* alterations between G-type adenomas and G-type adenocarcinomas, it is possible that *GNAS* alterations arise during gastric mucosal metaplasia, which serves as a precursor lesion. *GNAS* alterations are considered characteristic alterations in the carcinogenesis of G-type tumors in the duodenum.

The characteristics of the detected genomic alterations differed partially between those in previous reports and this study. In this study, *APC* and *KRAS* alterations were frequently detected, as reported earlier [15,16,17,18,19,20,21,22,23,24,25,37]; however, unlike previous reports, *TP53* alterations were rare in this study. Studies on the genomic analysis of duodenal tumors are scarce, with most of them using surgical specimens and focusing on stage II or higher cases. These studies reported high frequencies of *TP53*, *APC*, and *KRAS* alterations. The participants in the current study predominantly comprised patients with early lesions, including adenomas and early-stage cancers (Table S3: Clinicopathological characteristics of 127 adenocarcinomas), and the analysis revealed almost no *TP53* alterations (Figure S3: Genomic alteration plots of 158 duodenal epithelial neoplasms). Previous studies using endoscopic resection specimens have also demonstrated few *TP53* alterations [26,35]. The carcinogenesis mechanism of duodenal cancer appears to be similar to that of the adenoma–carcinoma sequence, one of the mechanisms of CRC carcinogenesis [38,39,40], which reflects the types of genomic alterations and their timing of occurrence.

**Figure 2:**
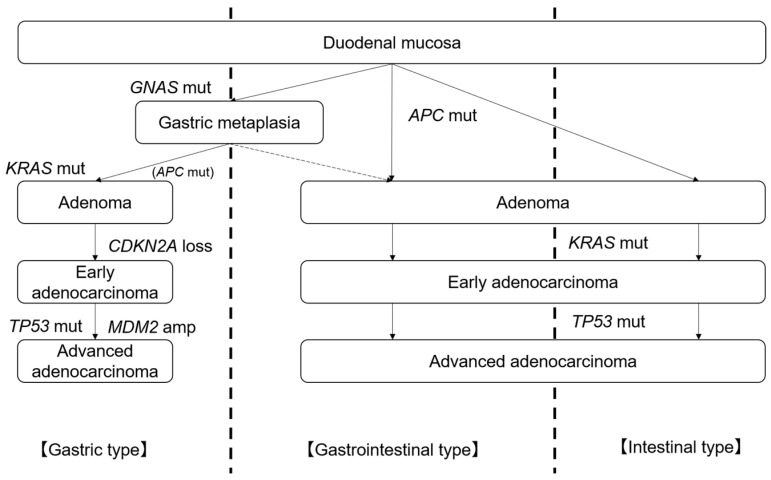
Predicted mechanism of duodenal tumor carcinogenesis. Left column: Carcinogenic mechanism of G-type tumors. Progression from gastric mucosal metaplasia to adenoma, early-stage adenocarcinoma, and advanced adenocarcinoma occurs through the development of *GNAS* alterations, *KRAS* alterations, *APC* alterations, *CDKN2A* loss, *TP53* alterations, and *MDM2* amplification. Central column: Carcinogenic mechanisms underlying GI-type tumors. There are pathways leading to *GNAS* alterations and *APC* alterations. Subsequently, *KRAS* and TP53 alterations occur, leading to progression through adenoma, early-stage adenocarcinoma, and advanced adenocarcinoma. Right column: Carcinogenesis mechanism of I-type tumors. Alterations in *APC*, *KRAS*, and *TP53* cause the progression of normal duodenal mucosa to adenoma, early-stage adenocarcinoma, and advanced adenocarcinoma.

On the basis of the current results and previously reported findings, we predicted the mechanism of duodenal tumor carcinogenesis (Figure 2). The mechanism of G-type tumor carcinogenesis is thought to proceed as follows. *GNAS* alterations in duodenal mucosal epithelial cells lead to gastric metaplasia. *KRAS* and *APC* mutations in metaplastic cells cause adenoma formation. *CDKN2A* loss subsequently results in early-stage adenocarcinoma. Further progression to advanced adenocarcinoma is expected via *TP53* mutations and *MDM2* amplification. The carcinogenic mechanism of type I tumors is thought to be similar to that of colorectal tumors. Adenomas develop when *APC* mutations occur in the duodenal mucosal epithelial cells. Early-stage adenocarcinomas develop because of *KRAS* mutations. *TP53* mutations are believed to drive the progression of advanced adenocarcinoma. The carcinogenic mechanism of GI-type tumors may originate from gastric metaplastic cells arising from *GNAS* mutations; however, it is believed that most cases progress to advanced adenocarcinoma when *KRAS* and *TP53* mutations occur in cells with *APC* mutations.

TMB-H was observed in 11% duodenal tumors. Previous reports indicated that approximately 10% of small intestinal adenocarcinomas exhibit TMB-H [41]. Similar to lung cancers driven by chemically induced genomic alterations or skin cancer caused by ultraviolet-induced genomic damage [42,43], one possible reason is chronic exposure to multiple digestive fluids, such as gastric acid, bile, and pancreatic juice, which are strong irritants. TMB-H tumors are good candidates for immune checkpoint inhibitors (ICIs). Therefore, testing for TMB-H status in advanced duodenal cancer is considered beneficial, as it may indicate that ICIs are a potential treatment option.

This study has some limitations. Because most specimens in this study were obtained by endoscopic resection, the tumor volume was low, resulting in approximately 20% of cases with low genomic content. Therefore, genomic alterations with low VAF may not have been detected because of the cut-off value. In the future, it will be necessary to trim small samples before sequencing. As most cases in this study were early-stage lesions, the genomic profile of early duodenal tumors was identified. However, owing to the low proportion of advanced cancers, these results may not adequately represent the overall genomic profile of duodenal cancer. Therefore, inclusion of a larger number of advanced cancer cases is necessary to enable a comprehensive genomic analysis of duodenal cancer across all stages.

## 5. Conclusions

In this study, TMB-H tumors were detected in >10% of duodenal tumors. Furthermore, results indicated that the pathogenesis of G-type duodenal tumors differs from that of other immunophenotypic tumors. These findings can help in understanding the genomic profiles of duodenal tumors, which are necessary for selecting appropriate treatment options.

## Figures and Tables

**Table 1:** Clinicopathological characteristics by histopathological type.

	Adenocarcinoma (*n* = 127)		Adenoma (*n* = 31)		*p*-Value
Sex					0.063 ^a^
Male	88	(69%)	16	(52%)	
Female	39	(31%)	15	(48%)	
Age					0.923 ^b^
Mean [95% CI], years	66.8	[64.7–68.9]	67.6	[64.4–70.9]	
Size					0.002 ^b^
Mean [95% CI], mm	31.8	[27.9–35.6]	21.7	[13.9–29.6]	
Resection method					1.000 ^a^
Endoscopic resection	123	(97%)	31	(100%)	
Surgical resection	4	(3%)	0	(0%)	
Location					0.423 ^a^
Superior	32	(25%)	9	(29%)	
Descending	60	(47%)	17	(55%)	
Horizontal	35	(28%)	5	(16%)	
Macroscopic classification					0.419 ^a^
I	22	(17%)	10	(32%)	
I + IIb	1	(1%)	0	(0%)	
IIa	91	(72%)	20	(65%)	
IIa + IIc	3	(2%)	1	(3%)	
IIc	8	(6%)	0	(0%)	
2	1	(1%)	0	(0%)	
3	1	(1%)	0	(0%)	
Immunophenotype					0.265 ^a^
Gastric	19	(15%)	4	(13%)	
Gastrointestinal	70	(55%)	13	(42%)	
Intestinal	38	(30%)	14	(45%)	
Gene alteration					
TMB-H	13	(10%)	5	(16%)	0.261 ^a^
*APC*	63	(50%)	10	(32%)	0.082 ^a^
*KRAS*	40	(31%)	10	(32%)	0.935 ^a^
*GNAS*	12	(9%)	5	(16%)	0.219 ^a^
*ERBB2*	5	(4%)	4	(13%)	0.075 ^a^
*CDKN2A*	7	(6%)	1	(3%)	0.511 ^a^
*ARID1A*	7	(6%)	0	(0%)	0.210 ^a^
*MDM2*	6	(5%)	1	(3%)	0.586 ^a^
*BRAF*	5	(4%)	0	(0%)	0.330 ^a^

^a^, Fisher exact test; ^b^, Mann–Whitney U test; CI, confidence interval; TMB-H, high tumor mutational burden.

**Table 2:** Clinicopathological characteristics by immunophenotype.

	Gastric (*n* = 23)		Gastrointestinal (*n* = 83)		Intestinal (*n* = 52)		*p*-Value
Sex							0.579 ^a^
Male	17	(74%)	52	(63%)	35	(67%)	
Female	6	(26%)	31	(37%)	17	(33%)	
Age							<0.001 ^b^
Mean [95% CI], years	75.2	[71.1–79.3]	65.8	[63.5–68.1]	65.2	[61.9–68.5]	
Size							0.019 ^b^
Mean [95% CI], mm	34.0	[24.0–44.1]	32.2	[27.4–37.1]	22.9	[17.1–28.7]	
Resection method							0.055 ^a^
Endoscopic resection	22	(96%)	83	(100%)	49	(94%)	
Surgical resection	1	(4%)	0	(0%)	3	(6%)	
Location							<0.001 ^a^
Superior	21	(91%)	12	(14%)	8	(15%)	
Descending	2	(9%)	44	(53%)	31	(60%)	
Horizontal	0	(0%)	27	(33%)	13	(25%)	
Macroscopic classification							0.002 ^a^
I	11	(49%)	13	(16%)	8	(15%)	
I + IIb	1	(4%)	0	(0%)	0	(0%)	
IIa	9	(39%)	62	(74%)	40	(77%)	
IIa + IIc	1	(4%)	3	(4%)	0	(0%)	
IIc	0	(0%)	5	(6%)	3	(6%)	
2	0	(0%)	0	(0%)	1	(2%)	
3	1	(4%)	0	(0%)	0	(0%)	
Histopathological type							0.265 ^a^
Adenocarcinoma	19	(83%)	70	(84%)	38	(73%)	
Adenoma	4	(17%)	13	(16%)	14	(27%)	
Gene alteration							
TMB-H	2	(9%)	12	(14%)	4	(8%)	0.440 ^a^
*APC*	7	(30%)	45	(54%)	21	(40%)	0.076 ^a^
*KRAS*	15	(65%)	25	(30%)	10	(19%)	<0.001 ^a^
*GNAS*	14	(61%)	3	(4%)	0	(0%)	<0.001 ^a^
*ERBB2*	0	(0%)	5	(6%)	4	(8%)	0.507 ^a^
*CDKN2A*	5	(22%)	2	(2%)	1	(2%)	0.004 ^a^
*ARID1A*	0	(0%)	5	(6%)	2	(4%)	0.664 ^a^
*MDM2*	7	(30%)	0	(0%)	0	(0%)	<0.001 ^a^
*BRAF*	1	(4%)	2	(2%)	2	(4%)	0.699 ^a^

^a^, Fisher exact test; ^b^, Kruskal–Wallis test; CI, confidence interval; TMB-H, high tumor mutational burden.

## Data Availability

All data generated or analyzed in this study are included in this paper and its Supplementary Materials. However, due to ethical restrictions, data beyond those mentioned above (e.g., individual data or detailed genomic alteration information) cannot be made publicly available.

## Supplementary Materials

The following supporting information can be downloaded at: https://www.mdpi.com/article/10.3390/cancers18132097/s1, Figure S1: Representative examples of each immunophenotype of duodenal epithelial neoplasms; Figure S2: Scoring system of genomic alteration; Figure S3: Genomic alteration plots of 158 duodenal epithelial neoplasms; Figure S4: Genomic alteration plots by histopathological type; Table S1: List of 160 cancer-related genes in the PleSSison-Rapid test; Table S2: List of 145 cancer-related genes and 11 fusions in the Rapid-Neo test; Table S3: Clinicopathological characteristics of 127 adenocarcinomas.

## Abbreviations

The following abbreviations are used in this manuscript:

CI	Confidence interval
CNAs	Copy number alterations
CRC	Colorectal cancer
DIN	DNA integrity number
FFPE	Formalin-fixed, paraffin-embedded
ICIs	Immune checkpoint inhibitors
IHC	Immunohistochemical
MSI-H	Microsatellite instability-high
PCR	Polymerase chain reaction
TMB-H	High tumor mutational burden
VAF	Variant allele frequency
VUS	Variant of unknown significance
