# Supplementary material for: Real-World Data of Comprehensive Genomic Profiles and Clinicopathological Characteristics of Duodenal Epithelial Neoplasms
Source: Cancers (Basel). 2026 Jun 28;18(13):2097. doi: 10.3390/cancers18132097 (PMC13360436; doi:10.3390/cancers18132097)
Supplement: Supplementary file 1 [file cancers-18-02097-s001.zip › Figure S1.pptx]

## Slide 1
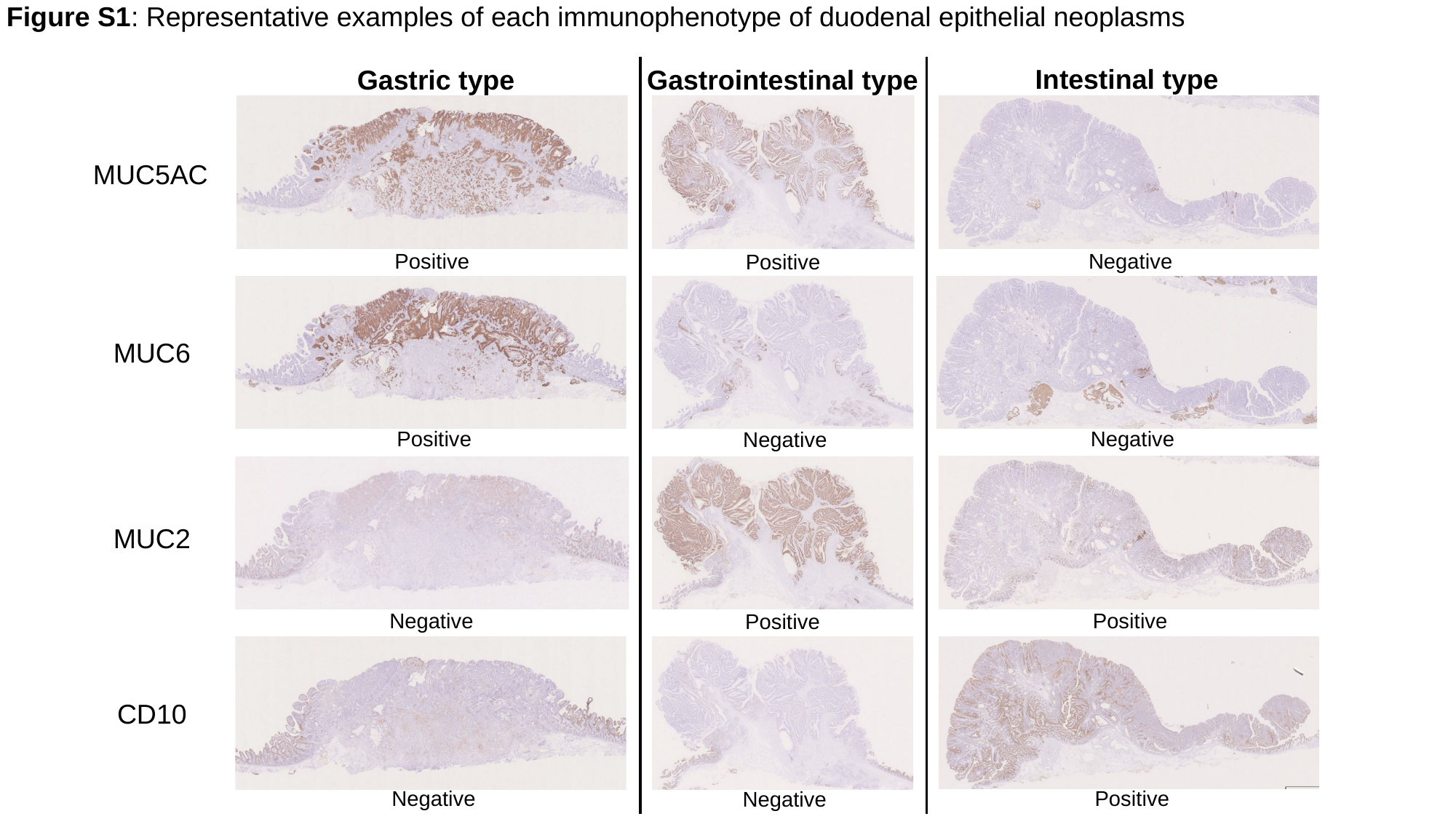

Figure S1: Representative examples of each immunophenotype of duodenal epithelial neoplasms
Intestinal type
Gastric type
Gastrointestinal type
MUC5AC
Positive
Negative
Positive
MUC6
Positive
Negative
Negative
MUC2
Negative
Positive
Positive
CD10
Negative
Positive
Negative
