# Supplementary material for: Real-World Data of Comprehensive Genomic Profiles and Clinicopathological Characteristics of Duodenal Epithelial Neoplasms
Source: Cancers (Basel). 2026 Jun 28;18(13):2097. doi: 10.3390/cancers18132097 (PMC13360436; doi:10.3390/cancers18132097)
Supplement: Supplementary file 1 [file cancers-18-02097-s001.zip › Figure S2.pptx]

## Slide 1
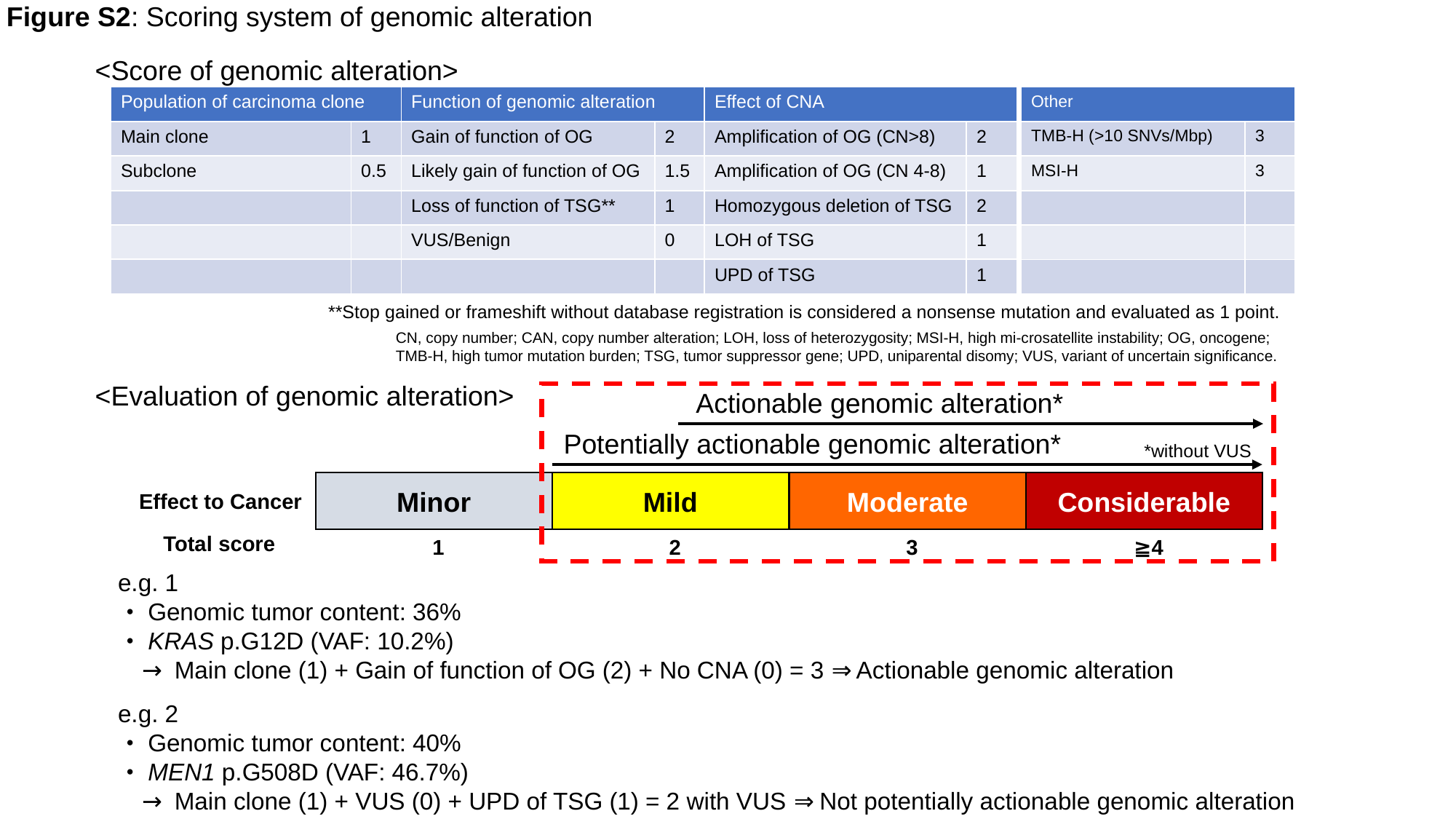

Figure S2: Scoring system of genomic alteration
<Score of genomic alteration>
| Population of carcinoma clone | | Function of genomic alteration | | Effect of CNA | |
| --- | --- | --- | --- | --- | --- |
| Main clone | 1 | Gain of function of OG | 2 | Amplification of OG (CN>8) | 2 |
| Subclone | 0.5 | Likely gain of function of OG | 1.5 | Amplification of OG (CN 4-8) | 1 |
| | | Loss of function of TSG\*\* | 1 | Homozygous deletion of TSG | 2 |
| | | VUS/Benign | 0 | LOH of TSG | 1 |
| | | | | UPD of TSG | 1 |
| Other | |
| --- | --- |
| TMB-H (>10 SNVs/Mbp) | 3 |
| MSI-H | 3 |
| | |
| | |
| | |
**Stop gained or frameshift without database registration is considered a nonsense mutation and evaluated as 1 point.
CN, copy number; CAN, copy number alteration; LOH, loss of heterozygosity; MSI-H, high mi-crosatellite instability; OG, oncogene;
TMB-H, high tumor mutation burden; TSG, tumor suppressor gene; UPD, uniparental disomy; VUS, variant of uncertain significance.
<Evaluation of genomic alteration>
Actionable genomic alteration*
Potentially actionable genomic alteration*
*without VUS
Minor
Mild
Moderate
Considerable
Effect to Cancer
Total score
1
2
3
≧4
e.g. 1
・Genomic tumor content: 36%
・KRAS p.G12D (VAF: 10.2%)
　→ Main clone (1) + Gain of function of OG (2) + No CNA (0) = 3 ⇒ Actionable genomic alteration
e.g. 2
・Genomic tumor content: 40%
・MEN1 p.G508D (VAF: 46.7%)
　→ Main clone (1) + VUS (0) + UPD of TSG (1) = 2 with VUS ⇒ Not potentially actionable genomic alteration
